# Preferred Materials and Methods Employed for Endodontic Treatment by Iranian General Practitioners

**Published:** 2015-03-18

**Authors:** Maryam Raoof, Negar Zeini, Jahangir Haghani, Saeedeh Sadr, Sakineh Mohammadalizadeh

**Affiliations:** a *Department of Endodontics, Dental School, Kerman University of Medical Sciences, Kerman, Iran; *; b * Oral and Dental Diseases Research Center, Kerman University of Medical Sciences, Kerman, Iran;*; c *Laboratory of Molecular Neuroscience, Neuroscience Research Center, Institute of Neuropharmacology, Kerman University of Medical Sciences, Kerman, Iran; *; d * Department of Oral and Maxillofacial Radiology, Dental School, Kerman University of Medical Sciences, Kerman, Iran; *; e * Endodontic Department, Hormozgan University of Medical Sciences, Bandar Abbas, Hormozgan, Iran; *; f * Instructor, Kerman University of Medical Sciences, Kerman, Iran*

**Keywords:** Apex Locator, Dentists, Endodontic Treatment, Iran, Obturation, Root Canal Therapy

## Abstract

**Introduction:** The aim of this study was to gather information on the materials and methods employed in root canal treatment (RCT) by general dental practitioners (GDPs) in Iran. **Methods and Materials:** A questionnaire was distributed among 450 dentists who attended the 53^th^ Iranian Dental Association congress. Participants were asked to consider demographic variables and answer the questions regarding the materials and methods commonly used in RCT. Descriptive statistics were given as absolute frequencies and valid percentages. The chi-square test was used to investigate the influence of gender and the years of professional activity for the employed materials and techniques.** Results:** The response rate was 84.88%. The results showed that 61.5% of the participants did not perform pulp sensitivity tests prior to RCT. Less than half of the general dental practitioners (47.4%) said that they would trace a sinus tract before starting the treatment. Nearly 16% of practitioners preferred the rubber dam isolation method. Over 36% of the practitioners reported using formocresol for pulpotomy. The combined approach of working length (WL) radiographs and electronic apex locators was used by 35.2% of the practitioners. Most of the respondents used K-file hand instruments for canal preparation and the technique of choice was step-back (43.5%), while 40.1% of respondents used NiTi rotary files, mostly ProTaper and RaCe. The most widely used irrigant was normal saline (61.8%). Calcium hydroxide was the most commonly used inter appointment medicament (84.6%). The most popular obturation technique was cold lateral condensation (81.7%) with 51% using zinc oxide-eugenol-based sealers.** Conclusions:** The majority of Iranian GDPs who participated in the present survey do not comply with quality guidelines of endodontic treatment.

## Introduction

Economic, functional, aesthetic and physiological advantages of saving natural teeth, have increased the importance of endodontic treatment. However, root canal treatment (RCT) is considered to be a tedious procedure for general dental practitioners (GDPs) [1]. It has been shown that more than 50% of teeth do not receive acceptable well-qualified RCT and approximately 30-50% of them develop some radiographic signs of apical periodontitis [2].

The outcome of endodontic therapy is highly dependent on maintenance of treatment standards [3]. However, several studies in different parts of the world, have reported the majority of dentists not being in compliance with quality assurance guidelines [4-8]. Moreover, epidemiological studies suggest that the endodontic failure rate is distinctly higher for teeth treated by non-specialist dentists [9]. Despite the importance of the subject, only one published study has been carried out in Iran that investigated the current opinions of the GDPs regarding fundamental aspects of routine endodontic treatment [10]. As the attitudes and approaches of GDPs toward endodontic therapy reflect the quality of the treatment and little information is available on how far the changes in endodontic technique have been incorporated into daily practice, the purpose of this study was to gather information on the materials and methods employed in RCT by GDPs in Iran.

## Materials and Methods

This cross-sectional study was approved by the Research Ethics Committee at Kerman University of Medical Sciences (Grant no.: k-92-286). The questionnaire for data gathering in this research was a modified version of some previous similar studies [1-3]. The structured questionnaire comprised of 20 questions with multiple-choice answers with an option for "other". Space was provided below the questions to add any additional comments, in case any treatment modification was not adequately covered by the given choices. The questionnaire included demographic information and questions about the stages, materials, and methods that are typically used in endodontic therapy.

To estimate the content validity index (CVI), six endodontists commented on each question. The CVI of each question was 0.9 to 1, which confirmed the validity of the questionnaire. A pilot study on 20 GDPs was conducted. Considering *α*=0.05 and *d*=0.04, the required minimal total sample size was calculated to be 384 subjects. For assessing test-retest reliability, the subjects were asked to re-answer a follow-up questionnaire 10 days later. 

The questionnaires were distributed among 450 GDPs participating in the 53^th^ Iranian Dental Association (IDA) congress in Tehran, May 2013. The participants were selected through simple random sampling technique. Respondents were assured of the anonymity and confidentiality of their responses. Two senior dental students were assigned handing out and collecting the questionnaires. Respondents were asked to return the completed questionnaires within half an hour. The questionnaire staff did not wait near the participants while they were answering questions.

**Table 1:** Demographic and professional data of the participants (*n*=382)

**Age (years)**	**Frequency N (%) **
**≤30**	169 (44.2)
**31-40**	103 (27)
**>40**	110 (28.8)
**Gender**	
**Female**	196 (51.3)
**Male**	186 (48.7)
**Practice experience (months)**	
**≤12**	57 (15)
**13-36**	85 (22.2)
**37-120**	70 (18.3)
**121-240**	59 (15.5)
**>240**	111 (29.0)
**Type of practice**	
**Private office**	218 (57.1)
**Community service**	108 (28.3)
**Both**	56 (14.6)

The data was processed using the SPSS software (SPSS version 17.0, SPSS, Chicago, IL, USA). For the analysis, the chi-square test was used and the level of significance was set at 0.05. The results were then calculated as absolute frequencies and valid percentages. The participants could have more than one answer.

## Results

A total of 382 completed questionnaires were collected, representing a response rate of 84.88%. The demographic characteristics of participants are shown in Table 1.

Only 38.5% of the participants reported performing pulp vitality tests before RCT and about one half of them would trace a sinus tracts routinely.

Cotton roll isolation was the most frequently used isolation method (75.5%). Moreover, 1.6% of the participating GDPs wouldn’t isolate the tooth before RCT. Rubber dam was routinely used by only 16.5% of the respondents.

Most of the respondents were using a cotton pellet moistened with formocresol, eugenol or calcium hydroxide (CH) while performing a pulpotomy. Only 16.8% reported that they use dry cotton pellet in this situation.

Regarding working length (WL) determination, over 60% of the respondents were using conventional radiographs, whereas 8.4% reported capturing digital radiographic images. About 35% used a combination of both radiography and electronic apex locators (EAL) (Table 2).

Over 43% of the respondents used step-back technique for preparation of the root canal system. Moreover, hand K-files and rotary instruments were used by 65.4% and 40.1% of GDPs, respectively. There was fairly equal distribution in the use of the rotary systems except for the ProTaper and RaCe systems with a reported usage of 19.1% and 14.1%, respectively. Also, there was almost an equal percentage of those who were using torque-controlled electric motors (20.9%) or air motors (20.4%) for rotary NiTi systems. However, almost half of the participants used hand files to shape the canals manually. For preflaring of the canal, almost half of the respondents used Gates Glidden drills while14.4% used rotary NiTi orifice shapers.

Normal saline was the most frequently used irrigating solutions (61.8%), whereas 42.9% of respondents used sodium hypochlorite (NaOCl). The most commonly used concentration of NaOCl was 0.5%. Most of the remainder of respondents used chlorhexidine (CHX) (Table 3).

**Table 2:** Method of working length determination

**Method**	**Frequency N (%)**
**Tactile sensation**	13 (3.5)
**Electronic apex locator (without radiography)**	63 (16.5)
**Conventional radiography**	240 (62.8)
**Paper cone**	16 (4.2)
**Digital radiography**	32 (8.4)
**Apex locator with radiography**	136 (35.7)

CH was the most commonly used intracanal medicament (used by 84.6% of respondents). The remaining practitioners used different formulations, including CHX, eugenol, formocresol and antibiotics. However, 6% did not use any medicament (Table 4).

The first choice of sealer was zinc oxide eugenol-based sealers (51%), followed by the resin-based sealers (44.5%). For the choice of obturation technique, the majority (81.7%) of participants used cold lateral condensation while vertical condensation was selected by 12% of the respondents.

There was an association between years of professional experience and the most appropriate answers (*P*=0.003). The proportion of participants who are in compliance with established quality assurance guidelines in endodontic procedures was the lowest among GDPs with more than 20 years of professional experience. None of the other variables affected the choice of materials and methods.

## Discussion

The current study evaluated the preference and selection of the materials, methods, and current trends used during RCT by a selected population of Iranian GDPs. We targeted GDPs for our study because epidemiological studies suggest that the failure rate is distinctly higher for teeth treated by non-specialist dentists [3-5]. Moreover, it seems that GDPs provide the majority of dental treatments in Iran.

Even though proper pulp testing is imperative to provide both a proper diagnosis and prognostic information [6], only 38.5% of the respondents perform pulp sensitivity tests prior to RCT.

Examination of sinus tracts should include tracing with gutta-percha cones to establish the origin of the lesion [7]. Unluckily, the present study revealed that less than half of the GDPs said that they would trace a sinus tract before starting the treatment.

According to the guidelines of the European Society of Endodontology (ESE), RCT procedures should be carried out only when the tooth is isolated by rubber dam [8], however, only 16.5% of GDPs reported using rubber dam. Anyway, this level of rubber dam usage is higher than the frequency reported in the surveys by Slaus and Bottenberg [9], Ravanshad *et al*. [1], Unal *et al*. [2], Al-Omari [10], and Kaptan *et al*. [11]. However, a survey amongst American GDPs showed that 59% of respondents always used rubber dam [12]. Practitioners may equate rubber dam use with extra cost, additional chair time, lack of adequate skills or training, underestimation of its benefits, absence of patient's acceptability or inadequate education in the undergraduate teaching curriculum [13].

**Table 3:** Root-canal irrigant of choice

**Irrigant**	**Frequency N (%)**
**NaOCl**	164 (42.9)
**Normal saline**	236 (61.8)
**Hydrogen peroxide**	21 (5.5)
**Chlorhexidine**	65 (17)
**Alcohol**	1 (0.3)
**Distilled water**	2 (0.5)

There was a significant relation between the use of rubber dam and the time elapsed after graduation. More recently graduated dental practitioners were following the standard of endodontic treatment better than earlier graduated GDPs. This is in accordance with the results of studies by Kapitán *et al*. [13] and Whitworth* et al*. [14] but does not match to the results reported by Jenkins *et al*. [15]. 

Regarding emergency pulpotomies, it is shown that pain relieving action of dry cotton pellet is as much as pellets moistened with Cresatin, camphorated monochlorophenol, eugenol, or saline. Concern has been expressed about formocresol, as it has been classified as a probable carcinogen [16]. Notwithstanding, Over 36% of the practitioners reported using formocresol for pulpotomies. The results emphasize the need for continuing dental education programs for practitioners to update their knowledge.

Successful outcome in endodontic treatment essentially depends on establishing a proper WL [17]. More than 60% of the GDPs participating in the current research reported using conventional radiography in determination of WL. On the other hand, new generations of EAL have powerful microprocessors and are able to give accurate readings of WL [18]. According to the existing data, the most precise determination of WL is in combination with radiographs and EALs. Almost 35% of the participants reported using this combination method. There seems to be a reticence to use apex locators within some other countries too [19-21]. The reason behind this hesitation might be attributed to paucity of continuing training courses and the resultant unfamiliarity of the GDPs with the equipment, device accuracy and the high equipment prices. However, the results of the present study are inconsistent with those of a study carried out in 2008 in Japan, in which more than 90% used an EAL to determine WL [22].

Nevertheless, a survey of endodontic practice was conducted in 2008 in Iran [1]. The authors stated that 84% of the practitioners used radiograph for determining the WL, and only 2.7% used EAL. Results of another survey conducted in 2012 in the same country, revealed that 45.2% of the GDPs used EALs [23]. A recent study also found that about 30% of the practitioners in Iran were using the combined approach of a WL radiography and an EAL during RCT of a single-rooted tooth while, 39.3% used this method in multi-rooted teeth [3]. In the present study, over 35% incorporated electronic measurement in WL determination. There seems to be an overall increasing trend toward using EAL in Iran. It may be due to the importance of reducing the multiple exposures to radiation and availability of accurate and reliable devices. However, to promote the use of EAL, an emphasis should be put upon increasing awareness about the advantages of this useful technology. An effective approach may be incorporating educational courses at the undergraduate and continuing education levels.

**Table 4:** Intracanal medicament of choice

**Medicament**	**Frequency N (%)**
**Calcium hydroxide**	323 (84.6)
**Chlorhexidine**	19 (5)
**Antibiotics**	4 (1)
**Eugenol**	16 (4.2)
**Formocresol**	11 (2.9)
**Corticosteroids**	2 (0.5)
**Nothing**	23 (6)

Digital radiography has important positive features such as reduction of radiation dose and the ability to manipulate the image [24]. It was disappointing that only 8.4% of the GDPs reported using digital radiography. The high price of the equipment might be a reason why most of GDPs do not use it.

The current study also found that a small number of the participants (3.5%) relied upon tactile sensation for estimation of WL. Although tactile sensation, may be beneficial in experienced hands, it has some noticeable limitations. The files may bind against the walls at any position along the canal, or may perforate apically. These drawbacks make this technique of WL determination unreliable [25].

The step-back method was used by 43.5% of the respondents. This technique used to be very popular in the 1960s and 1970s before introduction of crown-down hand and rotary instrumentation [26]. Preparation techniques, such as the crown-down and the passive step-back technique and rotary methods were not commonly used. This finding emphasizes on the need for continuing endodontic training courses for GDPs.

Most GDPs were using hand instrumentation and were not inclined to use more advanced engine driven techniques for shaping the root canal. It seems that new developments are slowly being incorporated into daily practice. Similarly, practitioners in Jordan and Denmark tend to use hand instruments and are not inclined to use more advanced engine-driven techniques for shaping of the root canal system [10, 19].

Several studies have shown the superiority of NiTi files over conventional instruments used for shaping the root canals [25]. The present study showed that 40.1% of respondents were using NiTi rotary files, mostly ProTaper and RaCe, for root canal preparation. This figure is similar to the results of another survey recently conducted in Iran [23], but higher than the reported results by some other studies [1, 19, 26]. Faster and simpler preparation of root canals might be the reason for general practitioners using rotary instruments so commonly.

Thorough debridement of the root canal system cannot be achieved merely by mechanical means. Therefore, the use of an antimicrobial irrigant is strongly recommended [27]. Despite the fact that the use of saline is not recommended as an irrigating solution, normal saline was the most popular root canal irrigant in the present study. NaOCl solution (0.5-5.25%) is suggested as the gold standard irrigant because of its profound antimicrobial and tissue-solving capacities [27], an opinion that was shared by only 42.9% of our respondents; almost the same as results reported by Ravanshad *et al*. [1]. The current findings do not mirror the findings of Whitten *et al*. [12] and Clarkson* et al*. [27] who reported that more than 70% of the respondents were using NaOCl as an irrigant, while in surveys by Jenkins* et al*. [15], NaOCl was not used routinely. It can be surmised that reluctance to wide use of NaOCl in the present study may be due to the respondents’ reticence to use the rubber dam system. The risk of forcing NaOCl through the apical foramen into the surrounding periapical tissues is another factor discouraging its further use.

CH is recommended as the gold standard intracanal medicament in RCT [28]. In the current survey, over 80% of the participants were using CH as an intracanal medicament. This figure is considerably more than the 7%-frequency reported in the study by Jenkins* et al*. in the UK [15], or the 11.5% and 9% usage rates in the studies conducted in Jordan and in the USA, respectively [10, 12]. However, in Flemish and Turkish studies, the percentages of respondents using CH were 64.6 and 61.5% [9, 11]. In the present survey, 6% of practitioners stated they did not use any intracanal medication. Similar results were observed in the study by Kaptan* et al*. in Turkey [11].

In the current study, the lateral condensation of gutta-percha significantly surpassed the other obturation techniques. It may be due to the fact that it is a relatively simple and versatile technique that does not require expensive equipment. Similar results were observed in many other studies [1, 9, 15, 19]. The reticence to choose some other obturation techniques including injection of warm gutta-percha, carrier-based techniques and continuous-wave method, might be due to possible mishaps during obturation, complexity of the techniques, relatively high initial cost and the need for more equipment compared to simplicity of the lateral compaction technique. On the other hand, in most universities lateral compaction is the principally taught obturation technique.

Although single-cone technique cannot reliably fill the root canal space in all dimensions and is not recommended [20], 4.2% reported using a single-cone technique.

It was interesting to note that 51% of the respondents were still using zinc oxide eugenol root canal sealers. This finding is consistent with the results of another study conducted in Iran [1], whereas in studies conducted in Flanders and Turkey, resin-based root canal sealers were the most popular ones [2, 9].

The respondents in this study attended a dental congress and may not be truly representatives of the general dental population in Iran. However, based on the findings of the current research, it is suggested that GDPs be properly and adequately instructed in the standard endodontic instruments, materials and techniques via different scientific courses, conferences and continuing training programs. Today post-graduate programs are designed and held in many countries including Iran. The basic principles for endodontics can be improved by increasing GDPs’ interest in pursuing post-graduate education.

## Conclusion

The data from this survey indicate that a noticeable number of dental practitioners who attended the 53^th^ Iranian Dental Association congress violated the basic standards of endodontic treatment. For example, over half of general dental practitioners are still using formocresol for pulpotomy and saline for irrigation, and most of them did not use rubber dam as an isolation method.
